# Same Phenotype in Children with Growth Hormone Deficiency and Resistance

**DOI:** 10.1155/2018/5902835

**Published:** 2018-04-15

**Authors:** Irene Ioimo, Carmen Guarracino, Cristina Meazza, Horacio M. Domené, Mauro Bozzola

**Affiliations:** ^1^University of Pavia, Piazzale Golgi 2, 27100 Pavia, Italy; ^2^Centro de Investigaciones Endocrinológicas “Dr. César Bergadá” (CEDIE) CONICET-FEI, División de Endocrinología, Hospital de Niños “Ricardo Gutiérrez”, Buenos Aires, Argentina; ^3^Onlus “Il Bambino e il suo Pediatra”, Galliate, Novara, Italy

## Abstract

By definition, about 2.5% of children show a short stature due to several causes. Two clinical conditions are characterized by serum IGF-I low levels, idiopathic GH deficiency (IGHD), and GH insensitivity (GHI), and the phenotypic appearance of these patients may be very similar. We studied two children with short stature and similar phenotypes. The first case showed frontal bossing, doll face, acromicria, and truncal obesity, with a GH peak <0.05 ng/ml after stimuli and undetectable serum IGF-I levels. After PCR amplification of the whole *GH1* gene, type IA idiopathic GHD was diagnosed. The second case had cranium hypoplasia, a large head, protruding forehead, saddle nose, underdeveloped mandible, and a micropenis. Basal GH levels were high (28.4 ng/ml) while serum IGF-I levels were low and unchangeable during the IGF-I generation test. Laron syndrome was confirmed after the molecular analysis of the GH receptor (*GHR*) gene. IGHD type IA and Laron syndrome is characterized by opposite circulating levels of GH, while both have reduced levels of IGF-I, with an overlapping clinical phenotype, lacking the effects of IGF-I on cartilage. These classical cases show the importance of differential diagnosis in children with severe short stature.

## 1. Introduction

By definition, about 2.5% of children show a short stature due to several causes, including familiar background, genetic and chromosomal abnormalities, hypothyroidism, and chronic diseases such as celiac and Crohn's disease [1].

Paediatric growth disease can also be secondary to growth hormone-insulin-like growth factor-I (GH-IGF-I) axis disorders, such as GH deficiency (GHD) or GH insensitivity (GHI), both characterized by serum low levels of IGF-I [2].

The first approach to a short child is clinical and based on the medical history and a physical examination focused on finding abnormal signs and dysmorphic features. When all other causes of short stature are excluded, the possibility of GHD arises. This is a rare condition with the prevalence approximately of 1 : 4,000 children [3]. The diagnosis must be supported by biochemical and neuroradiological results, that is, blunted GH response to at least two pharmacological stimuli, low concentration of IGF-I, and morphological abnormalities of the hypothalamus-pituitary axis, respectively [4]. Moreover, radiography of the left hand can indicate a delayed skeletal maturation of the patient.

Another condition with serum low IGF-I levels is Laron syndrome, the most common type of GH resistance. It is caused by homozygous mutation of the GH receptor (*GHR*) gene; the literature describes about 300–500 patients with different mutations [5–7]. Diagnosis of Laron syndrome must be supported by clinical and biochemical findings, such as low IGF-I levels, which do not increase after exogenous GH administration, elevated GH serum levels, and genetic studies.

We studied two children with similar phenotypes and severe short stature, but with different aetiology accounting for the short stature. Therefore, the aim of the present study was to identify the most reliable diagnostic parameters for differentiating children with comparable phenotypic characteristics.

## 2. Case 1

The first case was a South American small for gestational age girl, born in the 41st week of gestation from nonconsanguineous parents, previously described by Keselman et al. [8]. Neonatal auxological data are shown in Table 1.

**Figure 1:**
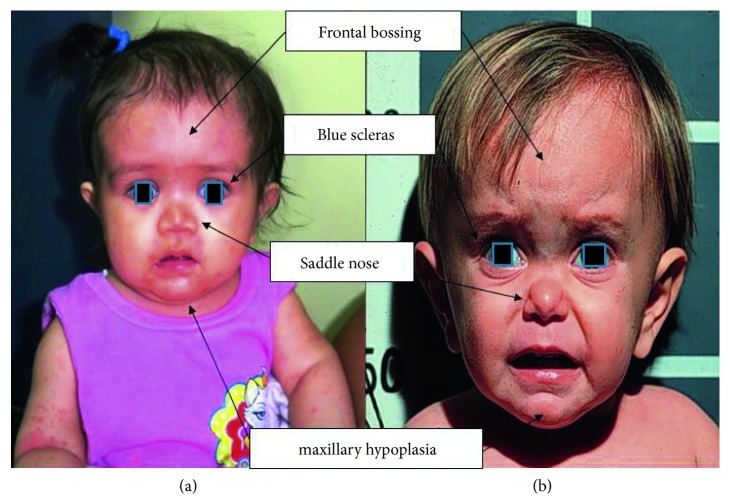
Comparison of facial features of the two patients: (a) the GHD type IA patient and (b) the Laron syndrome patient.

At the age of 10 months, she showed frontal bossing, doll face, acromicria, and truncal obesity (Figure 1). Psychomotor development was normal.

At 20 months of age, an endocrinological investigation was performed because of growth failure (Table 2). An arginine test showed a very low response of GH secretion, with a peak <0.05 ng/ml; IGF-I and IGF-binding protein-3 (IGFBP-3) levels were undetectable (<25 ng/ml and <0.5 *μ*g/ml, resp.  Table 3), while other pituitary hormone (thyroid and adrenal function) levels were normal (data not shown). A brain magnetic resonance imaging (MRI) showed a severe anterior pituitary hypoplasia.

As a congenital *GH* gene abnormality was hypothesized, a molecular characterization of *GH1* gene was performed; testing two genomic DNA samples by PCR amplification of the whole *GH1* gene did not yield any product, suggesting a *GH1* gene deletion. A subsequent analysis confirmed the presence of two deletions of 6.7 and 7.6 kb. Based on these results, type IA IGHD was diagnosed and the patient was started on rhGH replacement therapy (0.33 mg/kg/week).

## 3. Case 2

The second case was a Caucasian male, born at term by caesarean section because of fetopelvic disproportion from consanguineous parents (sons of first cousins). Neonatal auxological data are shown in Table 1. The perinatal period was complicated by transient hypoglycaemia occurring on the second day of life.

At 2.5 years, he showed cranium hypoplasia over the splanchnocranium, a large head compared with the rest of the body, a protruding forehead, depressed nasal bridge, underdeveloped mandible, and a micropenis (Figure 1). His auxological parameters are summarized in Table 2.

Because of growth retardation, he underwent an endocrinological evaluation. Basal GH levels were high (28.4 ng/ml) and increased after an arginine infusion (GH peak 67.1 ng/ml) suggesting a secretory hormone reserve. To exclude a biological inactivity of GH, an IGF-I generation test (three subcutaneous GH injections, 2 IU/day) was requested. The lack of increase in serum IGF-I values (basal levels: 48 ng/ml) after GH administration (peak: 51 ng/ml, Table 3) excluded a condition of GH bioinactivity suggesting a condition of GH insensitivity. High basal serum GH concentrations required a cerebral magnetic resonance imaging of the hypothalamus-pituitary region to exclude a pituitary neoplasia such as adenoma. All these results suggested a GH insensitivity syndrome. A molecular analysis of the *GHR* gene showed a W80X homozygous mutation of exon 5, of which his parents were both heterozygous carriers; this mutation usually causes a premature stop codon, resulting in a nonfunctional protein. Therefore, Laron syndrome was confirmed and the patient was administered a therapy with IGF-I (subcutaneous injections of doses up to 0.20 mg/kg/day), reaching a final stature of 135 cm.

## 4. Discussion

The phenotypic appearance of patients with complete GHD or GHI may be very similar since in both conditions a reduction or absence of the biological effects of GH is observed [12].

Patients with congenital isolated GHD have postnatal severe growth retardation in early infancy (during or after the first year of age) and a markedly short stature in adulthood. These children may show facial dysmorphisms such as craniofacial disproportion with hypoplasia of the midface, frontal bossing, doll face, and sparse and fine hair. Their phenotype also includes truncal obesity, acromicria, high-pitched voice, delayed skeletal maturation and dentition, and genital abnormalities like micropenis and delayed puberty [8, 13]. Similarly, patients with GHI syndrome show reduced birth length and intrauterine growth retardation and severe postnatal growth failure and short stature in adulthood. During childhood, patients generally show sparse hair, frontal bossing, saddle nose, shallow orbit, blue sclera, high-pitched voice, delayed dentition, genital abnormalities, sleep disorders, severe short stature, obesity, and delayed puberty [6, 13, 14].

As far as biochemical parameters are concerned, GHD is characterized by low-circulating GH and IGF-I levels, and IGF-II, IGFBP-3, and ALS reduction, reflecting their GH-dependent status [13].

In GHI, as in GHD, markedly reduced IGF-I, IGF-II, IGFBP3, and ALS levels are associated with normal or elevated serum GH levels, reflecting altered hypothalamic feedback [14].

As the two syndromes are characterized by opposite circulating levels of GH while both have reduced levels of IGF-I, the phenotypic appearance of GHI and GHD is most likely determined by the effect of IGF-I on cartilage. In fact, circulating IGF-I, primarily produced by the liver, and IGFBP-3 serum levels reflect endogenous GH secretion; GH also has direct effects independent of IGF-I, but systemic IGF-I is the main actor in the ossification process. Moreover, IGF-I bioactivity is modulated by circulating IGFBPs [15].

The GH-IGF-I axis plays a well-known role in the endochondral ossification of long bones and in determining stature, while craniofacial and somatic development is less dependent on GH action [16].

The first case shows typical characteristics of GHD, in particular, birth growth retardation with growth failure in the first year of life, an absence of psychomotor delay, truncal obesity, and facial dysmorphies such as midface hypoplasia, doll face, and frontal bossing. An MRI showed only an anterior pituitary hypoplasia, while those for patients with genetic GHD generally reveal a normal or hypoplastic pituitary gland without abnormalities of the hypothalamus, pituitary dystopia, agenesis of the pituitary gland, or thinning or interruption of the pituitary stalk [17].

Considering the severely short stature, defined as a height more than 3 SD below the mean, it is mandatory to consider investigating for GHD [18, 19]. We therefore measured baseline values of IGF-I, GH, and other pituitary hormones and evaluated GH secretion after two pharmacological stimuli. Our patient had undetectable baseline and peak values of GH, low serum levels of IGF-I, normal levels of other pituitary hormones, and low serum levels of IGFBP-3, compatible with isolated GHD. To determine the type of GHD, we also performed a genetic analysis that allowed us to diagnose type IA GHD; therefore, substitution therapy was initiated with rhGH. The literature reports a genotype/phenotype correlation in patients with IGHD [20]; children with type IA IGHD may have birth short length and neonatal hypoglycaemia along with undetectable GH concentrations with severe growth failure in the first 6 months. In fact, our patient began to show a worsening of growth in the first six months.

In the second case, the patient had a severe growth deficit with typical facial dysmorphies and elevated GH serum values. We assumed a GHI and an assay for basal IGF-I and IGFBP-3 levels were performed, with results below the normal range. Growth hormone insensitivity is a group of pathologies characterized by short stature associated with normal or elevated GH concentrations. Primary GH insensitivity is caused by genetic disorders involving GHR or its downstream mediators such as *STAT5b*, the IGF-I/IGFBP3-stabilized *acid labile subunit (ALS)*, *IGF-I*, or the *IGF-I receptor* gene [21]. The most common type is Laron syndrome, caused by homozygous mutation of the *GHR* gene. In other cases, the IGF-I deficiency may be secondary to chronic or inflammatory diseases or to malnutrition. Although in all cases there is a low-circulating level of IGF-I, the clinical phenotypes and biochemical characteristics within the GH-resistant patients group vary considerably from short stature to extreme failure growth.

Both of our patients showed an overlapping clinical phenotype due to the absence of effects of IGF-I on cartilage and an MRI without large abnormalities but contrasting biochemical data, particularly for GH values. Therefore, these classical cases of IGHD and Laron syndrome underline the importance of investigating severe short stature even in the absence of other symptoms in the neonatal age and without alteration to the MRI.

## Figures and Tables

**Table 1:** Neonatal auxological parameters of the two patients.

Diagnosis	Gestational age (weeks)	Birth weight (kg)	Head circumference (cm)	Birth length (cm)	Target height (cm)
GHD type IA	41	2.480 (−2.55 SDS)	34.1 (−0.42 SDS)	44.0 (−3.64 SDS)	153.5
Laron syndrome	39	3.350 (−0.30 SDS)	35.0 (0.18 SDS)	46.0 (−2.38 SDS)	163.5

Auxological parameters are expressed in SDS according to Bertino et al. [9].

**Table 2:** Auxological parameters of the two patients at the first endocrinological evaluation.

Diagnosis	Age (yrs.)	Weight (SDS)	Head circumference (SDS)	Height (SDS)	BMI (SDS)
GHD type IA	0.8	−3.60	−2.00	−5.09	−7.12
Laron syndrome	1.7	−4.60	−1.40	−6.10	0.12

Auxological parameters are expressed in SDS according to Cole et al. [10] and WHO charts [11].

**Table 3:** Biochemical parameters of the two patients at the time of diagnosis.

Diagnosis	Basal GH (ng/ml)	GH peak (ng/ml) after arginine	Basal IGF-I (ng/ml)	IGF-I peak (ng/ml) after GH	IGFBP-3 (*μ*g/ml)
GHD type IA	<0.05	<0.05	<25.0	n.a.	<0.5
Laron syndrome	28.4	67.1	48.0	51.0	0.5

n.a.: not available.

## References

[1] Stanley T. (2012). Diagnosis of growth hormone deficiency in childhood. *Current Opinion in Endocrinology, Diabetes and Obesity*.

[2] Savage M. O., Burren C. P., Rosenfeld R. G. (2010). The continuum of growth hormone-IGF-I axis defects causing short stature: diagnostic and therapeutic challenges. *Clinical Endocrinology*.

[3] Vimpani G. V., Vimpani A. F., Lidgard G. P., Cameron E. H., Farquhar J. W. (1977). Prevalence of severe growth hormone deficiency. *British Medical Journal*.

[4] Rogol A. D., Hayden G. F. (2014). Etiologies and early diagnosis of short stature and growth failure in children and adolescents. *Journal of Pediatrics*.

[5] Murray P. G., Clayton P. E., De Groot L. J., Chrousos G., Dungan K. (2000). Disorders of growth hormone in childhood. *Endotext*.

[6] Laron Z. (2015). Lessons from 50 years of study of Laron syndrome. *Endocrine Practice*.

[7] Janecka A., Kołodziej-Rzepa M., Biesaga B. (2016). Clinical and molecular features of Laron syndrome, a genetic disorder protecting from cancer. *In Vivo*.

[8] Keselman A., Scaglia P. A., Rodríguez Prieto M. S. (2012). Type IA isolated growth hormone deficiency (IGHD) consistent with compound heterozygous deletions of 6.7 and 7.6 Kb at the GH1 gene locus. *Arquivos Brasileiros de Endocrinologia & Metabologia*.

[9] Bertino E., Spada E., Occhi L. (2010). Neonatal anthropometric charts: the Italian neonatal study compared with other European studies. *Journal of Pediatric Gastroenterology and Nutrition*.

[10] Cole T. J., Freeman J. V., Preece M. A. (1995). Body mass index reference curves for the UK, 1990. *Archives of Diseases in Childhood*.

[11] WHO Multicentre Growth Reference Study Group (2006). WHO child growth standards based on length/height, weight and age. *Acta Paediatrica*.

[12] Laron Z., Klinger B. (1994). Laron syndrome: clinical features, molecular pathology and treatment. *Hormone Research*.

[13] Roberts C. T., Rosenfeld R. G. (1999). *The IGF System: Molecular Biology, Physiology, and Clinical Applications*.

[14] Ghosh S., Goswami S., Chowdhury S. (2012). Growth hormone insensitivity syndrome: a sensitive approach. *Indian Journal of Endocrinology and Metabolism*.

[15] Barreca A., Bozzola M., Cesarone A. (1998). Short stature associated with high circulating insulin-like growth factor (IGF)-binding protein-1 and low circulating IGF-II: effect of growth hormone therapy. *Journal of Clinical Endocrinology and Metabolism*.

[16] Singleton D. A., Buschang P. H., Behrents R. G., Hinton R. J. (2006). Craniofacial growth in growth hormone-deficient rats after growth hormone supplementation. *American Journal of Orthodontics and Dentofacial Orthopedics*.

[17] Di Iorgi N., Morana G., Allegri A. E. M. (2016). Classical and non-classical causes of GH deficiency in the paediatric age. *Best Practice & Research: Clinical Endocrinology and Metabolism*.

[18] Grimberg A., DiVall S. A., Polychronakos C. (2016). Guidelines for growth hormone and insulin-like growth factor-I treatment in children and adolescents: growth hormone deficiency, idiopathic short stature, and primary insulin-like growth factor-I deficiency. *Hormone Research in Paediatrics*.

[19] Growth Hormone Research Society (2000). Consensus guidelines for the diagnosis and treatment of growth hormone (GH) deficiency in childhood and adolescence: summary statement of the GH research society. *Journal of Clinical Endocrinology and Metabolism*.

[20] Alatzoglou K. S., Dattani M. T. (2012). Phenotype-genotype correlations in congenital isolated growth hormone deficiency (IGHD). *Indian Journal of Pediatrics*.

[21] Rosenbloom A. L. (2008). Physiology of growth. *Annales Nestlé*.

